# Mortality rate of acute kidney injury in SARS, MERS, and COVID-19 infection: a systematic review and meta-analysis

**DOI:** 10.1186/s13054-020-03134-8

**Published:** 2020-07-16

**Authors:** Yih-Ting Chen, Shih-Chieh Shao, Edward Chia-Cheng Lai, Ming-Jui Hung, Yung-Chang Chen

**Affiliations:** 1 Department of Nephrology, Department of Internal Medicine, Keelung Chang Gung Memorial Hospital, Keelung, Taiwan; 2 Institute of Public Health, School of Medicine, National Yang Ming University, Taipei, Taiwan; 3 School of Pharmacy, Institute of Clinical Pharmacy and Pharmaceutical Sciences, College of Medicine, National Cheng Kung University, Tainan, Taiwan; 4 Department of Pharmacy, Keelung Chang Gung Memorial Hospital, Keelung, Taiwan; 5 College of Medicine, Chang Gung University, Taoyuan, Taiwan; 6 Section of Cardiology, Department of Internal Medicine, Keelung Chang Gung Memorial Hospital, Keelung, Taiwan; 7 Kidney Research Center, Department of Nephrology, Linkou Chang Gung Memorial Hospital, Taoyuan, Taiwan; 8 Community Medicine Research Center, Keelung Chang Gung Memorial Hospital, Keelung, Taiwan; 9 Division of Nephrology, Department of Medicine, Keelung Chang Gung Memorial Hospital, No. 222, Maijin Rd., Anle Dist., Keelung, Taiwan

**Keywords:** Acute kidney injury, Mortality, COVID-19, MERS, SARS

Acute kidney injury (AKI), a predictor for poor clinical outcomes, has been reported as a severe complication of different coronavirus infections, including novel coronavirus disease 2019 (COVID-19) [1]. COVID-19 is considered more contagious than previous coronavirus infections, e.g., severe acute respiratory syndrome (SARS) and Middle East respiratory syndrome (MERS) [2], but comparisons of mortality rates from AKI among these three coronavirus infections remain uninvestigated. We therefore conducted a systematic review and meta-analysis comparing the mortality rate in patients with SARS, MERS, and COVID-19 who developed AKI.

A systematic search of PUBMED and EMBASE from inception to June 5, 2020, included the keywords “coronavirus”, “COVID-19”, “MERS”, “SARS”, “acute kidney injury”, “prognosis”, and “mortality” with suitable MeSH terms to identify observational studies of relevance, e.g., case reports, case series, cross-sectional studies, and cohort studies. Reference lists of included, published, systematic reviews identified in the search were screened for additional studies. We excluded conference abstracts, review articles, or studies without reports of AKI mortality. Two reviewers (YTC, SCS) screened titles and abstracts of search results for relevance and individually and independently assessed the full texts of selected results. The final list of included studies was derived by discussion and unanimous agreement from both authors. Statistical analyses were performed using MedCalc for Windows, version 15.0 (MedCalc Software, Ostend, Belgium). We report the mortality rate from AKI in SARS, MERS, and COVID-19 infections as proportions with 95% confidence interval (CI) based on random effects model, represented by forest plot. We detected heterogeneity among studies using the Cochran Q test, with *p* value < 0.10 indicating significant heterogeneity, and calculated *I*^2^ statistic to determine the proportion of total variation in study estimates attributable to heterogeneity.

After screening 97 records in total, we excluded 74 articles (15 duplicates, 11 irrelevant to study question, 1 conference abstract, 5 review articles and 42 lacking data on AKI mortality). Our final analysis included 23 articles comprising 4, 3 and 16 on SARS, MERS and COVID-19 infection, respectively. Demographic data for included articles are presented in Table 1. Overall, mortality in patients with SARS, MERS and COVID-19 infection, and developing AKI, was 77.4% (95%CI: 64.7–88.0). We found the mortality rate of AKI was highest in SARS (86.6%; 95%CI: 77.7–93.5), followed by COVID-19 (76.5%; 95%CI: 61.0–89.0) and MERS (68.5%; 95%CI: 53.8–81.5). There was no evidence of statistical heterogeneity among studies reporting AKI mortality in SARS (I2: 0.0%, *p* = 0.589) and MERS (I2: 0.0%, *p* =v0.758), but there was for COVID-19 infection (I2: 97.0%, *p* < 0.001) (Fig. 1).

**Table 1:** Study characteristics

Author and year	Country/city	AKI male (%)	AKI age (median)	Settings	Total case numbers	AKI case numbers	Baseline serum creatinine (mg/dL)	RRT/AKI case (%)	AKI mortality (%)	Overall mortality (%)
**SARS**										
Huang 2005 [3]	Taiwan/Taipei	77	65*	Hospitalization	78	13	1.20	38	77	19
Wu 2004 [4]	Taiwan/Taipei	50	58*	Hospitalization	2	2	1.05	NA	100	100
Chu 2005 [5]	China/Hong Kong	69	54	Hospitalization	536	36	1.06	28	92	14
Choi 2003 [6]	China/Hong Kong	NA	NA	Hospitalization	267	15	NA	NA	87	12
**MERS**										
Saad 2014 [7]	Saudi Arabia	NA	NA	Hospitalization	70	30	NA	NA	70	60
Alsaad 2017 [8]	Saudi Arabia	100	33	Intensive care unit	1	1	NA	0	100	100
Cha 2015 [9]	Korea	63	73*	Hospitalization	30	8	1.60	38	63	17
**COVID-19**										
Alberici 2020 [10]	Italy/Brescia	67	58*	Kidney transplantation/hospitalization	20	6	3.13	17	17	25
Hirsch 2020 [11]	USA/New York	64	69	Hospitalization	5449	1993	1.24	14	35	16
Lei 2020 [12]	China/Wuhan	NA	NA	Hospitalization	34	2	NA	NA	100	21
Chen 2020 [13]	China/Wuhan	NA	NA	Hospitalization	274	29	NA	10	97	41
Deng 2020 [14]	China/Wuhan	NA	NA	Hospitalization	225	20	NA	NA	100	48
Wang 2020 [15]	China/Wuhan	NA	NA	Hospitalization	107	14	NA	NA	100	18
Yang 2020 [16]	China/Wuhan	NA	NA	Hospitalization	52	15	NA	60	80	62
Gopalakrishnan 2020 [17]	USA	100	49	Hospitalization	1	1	1.00	100	100	100
Suwanwongse 2020 [18]	USA/New York	100	88	Hospitalization	1	1	1.16	0	0	0
Banerjee 2020 [19]	UK/London	25	59*	Kidney transplantation/hospitalization	7	4	2.54	75	25	14
Zhou 2020 [20]	China/Wuhan	NA	NA	Hospitalization	191	28	NA	36	96	28
Wang 2020 [21]	China/Wuhan	NA	NA	Hospitalization	339	27	NA	NA	63	19
Richardson 2020 [22]	USA/New York	NA	NA	Hospitalization	2351	523	NA	15	66	20
Wang 2020 [23]	China/Wuhan	NA	NA	Intensive care unit	344	86	NA	10	93	39
Ruan 2020 [24]	China/Wuhan	NA	NA	Hospitalization	150	23	NA	22	91	45
Cao 2020 [25]	China/Wuhan	NA	NA	Hospitalization	102	20	NA	30	75	17

*AKI* acute kidney injury, *NA* not available, *RRT* renal replacement therapy

*Age was represented by the mean value

**Fig. 1:**
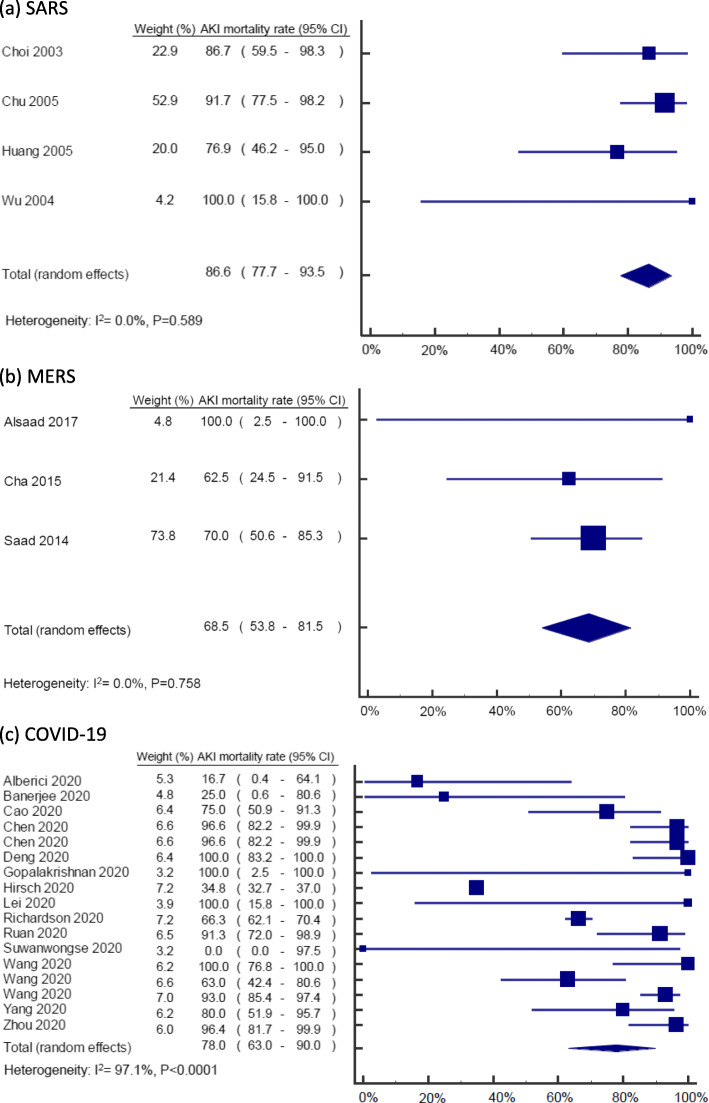
Forest plot of AKI mortality in coronavirus infections from included studies: **a** SARS, **b** MERS, and **c** COVID-19

The present analyses indicate AKI as a poor prognosis factor in coronavirus infections, whereby AKI mortality in COVID-19 is higher than MERS but lower than SARS infections. Possible mechanisms of higher AKI mortality following coronavirus infections are multifactorial (e.g., severe sepsis-related multi-organ failure, direct kidney involvement, and acute respiratory distress syndrome) [26–28], although comparative pathogenesis of kidney involvement among the three infections remains unclear.

To our best knowledge, this is the first systematic review exploring AKI mortality of different coronavirus infections. However, we should be cautious about interpreting causal relationships between coronavirus infections and AKI, given the nature of observational data. Also, clinical heterogeneity between studies should be noted; for example, various healthcare systems of included studies may produce different AKI mortality rates. Coronaviruses are unlikely to be eliminated in the near future, and our synthesis indicates that AKI secondary to coronavirus infection may contribute to higher mortality. Hence, in the current exceptional pandemic, first-line healthcare providers should recognize the importance of timely detection of AKI and consider all available treatment options for maintenance of kidney functions to prevent death in COVID-19 patients [29].

## Data Availability

Not applicable.

## Abbreviations

AKI: Acute kidney injury
CI: Confidence interval
COVID-19: Coronavirus disease 2019
MERS: Middle East respiratory syndrome
SARS: Severe acute respiratory syndrome
